# Autochthonous Outbreak and Expansion of Canine Visceral Leishmaniasis, Uruguay

**DOI:** 10.3201/eid2303.160377

**Published:** 2017-03

**Authors:** Dinora Satragno, Paula Faral-Tello, Bruno Canneva, Lorenzo Verger, Alejandra Lozano, Edgardo Vitale, Gonzalo Greif, Carlos Soto, Carlos Robello, Yester Basmadjián

**Affiliations:** Universidad de la República, Montevideo, Uruguay (D. Satragno, B. Canneva, L. Verger, A. Lozano, E. Vitale, C. Soto, C. Robello, Y. Basmadjián);; Instituto Pasteur, Montevideo (P. Faral-Tello, G. Greif, C. Robello)

**Keywords:** *Leishmania infantum*, Uruguay, canine visceral leishmaniasis, dogs, sand flies, vector-borne infections, zoonoses, protozoa, parasitic diseases, parasites

## Abstract

We report an outbreak of canine visceral leishmaniasis in Uruguay. Blood specimens from 11/45 dogs tested positive for *Leishmania* spp. Specimens of *Lutzomyia longipalpis* sand flies were captured; typing revealed *Leishmania infantum*. Our findings document an expansion of visceral leishmaniasis to southern South America and risk for vectorborne transmission to humans.

Visceral leishmaniasis (VL) is a zoonotic disease caused by flagellated protozoa of the genus *Leishmania* and transmitted by sand flies belonging to the Phlebotominae subfamily; those of the *Lutzomia longipalpis* species are the main vectors. VL affects humans and canids; canids are identified as the main reservoir of the parasite (*1*). This zoonosis has been endemic in northeastern Brazil for several centuries, but it has been recently expanding to southern areas of the South American continent (*2*–*4*). In 2010, the presence of the vector *L. longipalpis* sand flies was recorded for the first time in Uruguay (*5*); the right environmental conditions, the presence of competent sand fly vectors, and the constant appearance of new cases of canine and human leishmaniasis in border countries have made Uruguay susceptible to VL transmission (*5*).

In 2015, we performed a house-by-house survey in Arenitas Blancas (31°25.000′S, 58°00.066′W) in Salto, Uruguay. We included 49 dogs in the survey. Whole-blood samples from 11 (22%) tested positive for *Leishmania* spp. with 2 different diagnostic kits, TR DPP (Bio-Manguinhos, Rio de Janeiro, Brazil) and Speed Leish K (Virbac, Carros, France), both of which detect antibodies raised against *Leishmania* antigens in whole blood, plasma, or serum by immunochromatographic methods. Among the dogs whose specimens tested positive, 8 showed the common clinical signs of skin lesions, fever, weight loss, and eye lesions; 3 were asymptomatic. Dogs whose specimens tested positive came from 9 different houses in the same neighborhood (Figure, panel A); of these, 2 dogs had never traveled outside their residence, and in 2 other cases, both dam and offspring were infected. Three dogs came from breeding kennels, and the rest were born in Arenitas Blancas.

**Figure:**
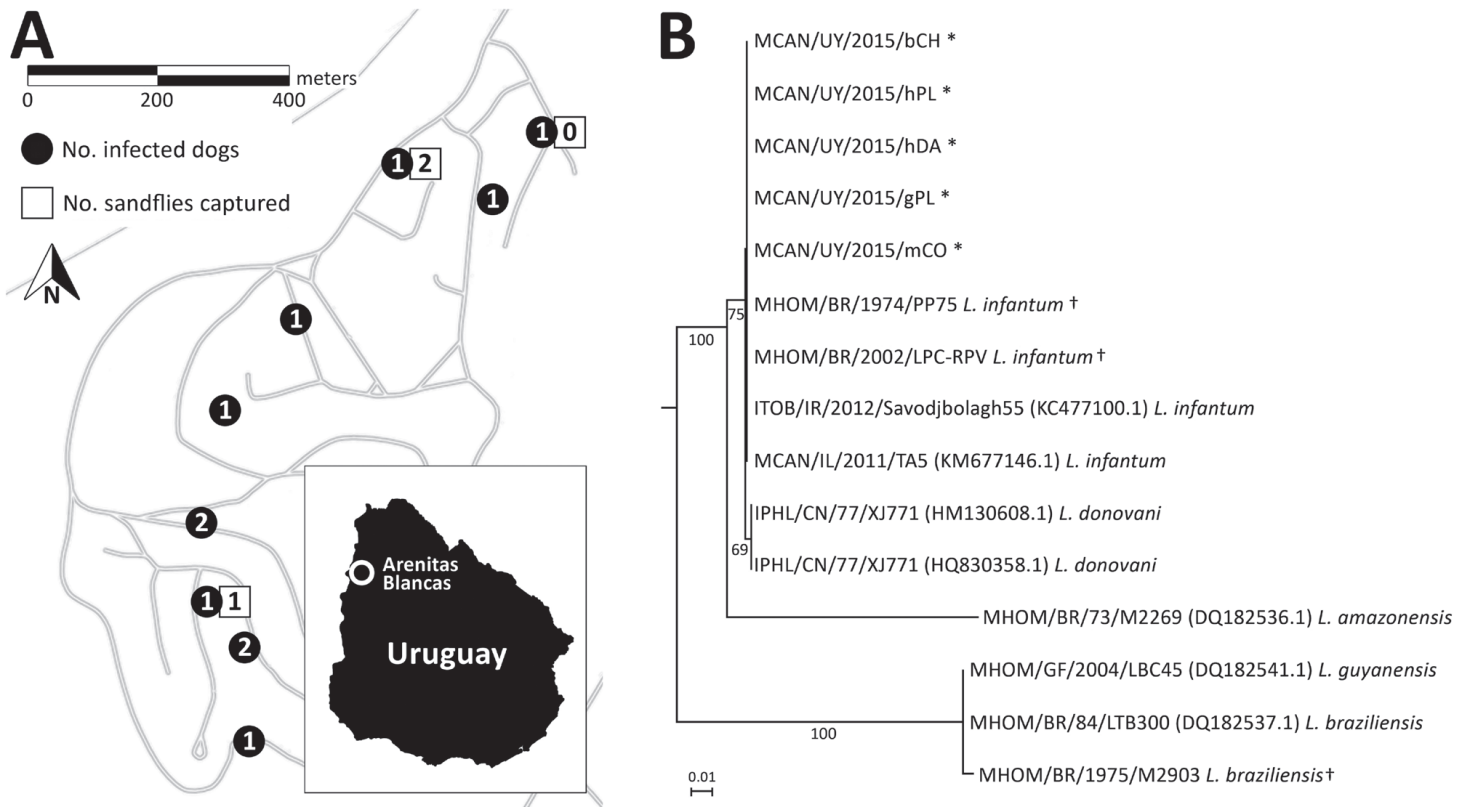
Survey of *Leishmania* spp. infection in dogs in Arenitas Blancas, Salto, Uruguay. A) Surveyed area in the locality of Arenitas Blancas in Salto, Uruguay. White squares represent the location of *Lutzomia longipalpis* sand fly captures, and black circles represent domiciles in which infected dogs were found; numbers indicate number of *Leishmania* spp.–infected sand flies or dogs at that location. B) Neighbor-joining phylogenetic tree obtained from the analysis of *Leishmania* internal transcribed spacer 1 sequences from tissue samples of infected dogs. Bootstrap values are represented at the nodes of major branches. Scale bar indicates nucleotide substitutions per site. *Sequences obtained from infected dog samples. †Reference strains sequenced by the authors.

We performed lymph node biopsies and bone marrow aspiration in dogs whose specimens tested positive; we also confirmed infection by direct observation of amastigotes in stained slide smears of aspirates. After extracting DNA from tissue samples by using the Quick-DNA Universal kit (Zymo Research, Irvine, California, USA), we performed PCR and sequencing of the ribosomal internal transcribed spacer 1 (*6*) to achieve typing of *Leishmania* spp. at the species level. We aligned and analyzed the sequences by using MAFFT software (*7*); the neighbor-joining phylogenetic tree obtained from the analysis showed that sequences identified from our samples group together with sequences belonging to *L. infantum* reference strains that we sequenced, as well as with sequences obtained from GenBank (Figure, panel B). Accession numbers and percentage of identity of the sequences obtained from GenBank are *L. infantum*, KM677146.1 and KC477100.1 (100%); *L. donovani*, HM130608.1 and HQ830358.1 (99%); *L. amazonensis*, DQ182536.1 (86%); *L. guyanensis*, DQ182541.1 (81%); and *L. braziliensis*, DQ182537.1 (81%).

To verify that the complete domestic cycle of *Leishmania* spp. was taking place in the affected area, we placed CDC Miniature Light Traps (John W. Hock Company, Gainesville, FL, USA) in domiciles in which affected dogs had been found. All sampling was peridomestic and consisted of 13 traps placed overnight on 3 different nights; sampling resulted in collection of 3 sand flies, 1 male and 2 female. Using observational analysis, we identified the collected samples as *L. longipalpis*; this result was confirmed by PCR with species-specific primer LiCac (*8*,*9*). Furthermore, we performed PCR amplification with *Leishmania*-specific primers AJS1 and DeB8 (*8*) using sand fly DNA as a template. A PCR product of 300 bp from one of the sand flies was amplified and sequenced and showed *Leishmania* DNA in the vector (data not shown).

In summary, we describe an autochthonous outbreak of canine VL in Uruguay. The reported cases represent the expansion of VL to southern areas of the continent; the evidence shows that *L. infantum* is the parasite responsible for the outbreak in both canine hosts and a sand fly vector. The presence of competent vectors in the area constitutes a risk for the human population. Further work is needed to implement effective measures to control the extension of cases. It is also mandatory to improve surveillance of the vector and expand surveillance to other wild and domestic potential hosts. Finally, efforts should be made to prevent new cases of human VL in Uruguay.

## References

[1] Ashford DA, David JR, Freire M, David R, Sherlock I, Eulálio MC, et al. Studies on control of visceral leishmaniasis: impact of dog control on canine and human visceral leishmaniasis in Jacobina, Bahia, Brazil. Am J Trop Med Hyg. 1998;59:53–7. 9684628 10.4269/ajtmh.1998.59.53

[2] Barrio A, Parodi CM, Locatelli F, Mora MC, Basombrío MA, Korenaga M, et al. *Leishmania infantum* and human visceral leishmaniasis, Argentina. Emerg Infect Dis. 2012;18:354–5. 10.3201/eid1802.110924 22305425 PMC3310450

[3] Gould IT, Perner MS, Santini MS, Saavedra SB, Bezzi G, Maglianese MI, et al. [Visceral leishmaniasis in Argentina. Cases notification and distribution of vectors (2006-2012)]. Medicina (B Aires). 2013;73:104–10. 23570757

[4] Maia-Elkhoury AN, Alves WA, Sousa-Gomes ML, Sena JM, Luna EA. Visceral leishmaniasis in Brazil: trends and challenges. Cad Saude Publica. 2008;24:2941–7. 10.1590/S0102-311X2008001200024 19082286

[5] Salomón OD, Basmajdian Y, Fernández MS, Santini MS. *Lutzomyia longipalpis* in Uruguay: the first report and the potential of visceral leishmaniasis transmission. Mem Inst Oswaldo Cruz. 2011;106:381–2. 10.1590/S0074-02762011000300023 21655832

[6] Schönian G, Nasereddin A, Dinse N, Schweynoch C, Schallig HD, Presber W, et al. PCR diagnosis and characterization of *Leishmania* in local and imported clinical samples. Diagn Microbiol Infect Dis. 2003;47:349–58. 10.1016/S0732-8893(03)00093-2 12967749

[7] Katoh K, Standley DM. MAFFT multiple sequence alignment software version 7: improvements in performance and usability. Mol Biol Evol. 2013;30:772–80. 10.1093/molbev/mst010 23329690 PMC3603318

[8] Smyth AJ, Ghosh A, Hassan MQ, Basu D, De Bruijn MH, Adhya S, et al. Rapid and sensitive detection of *Leishmania* kinetoplast DNA from spleen and blood samples of kala-azar patients. Parasitology. 1992;105:183–92. 10.1017/S0031182000074096 1333582

[9] Lins RM, Oliveira SG, Souza NA, de Queiroz RG, Justiniano SC, Ward RD, et al. Molecular evolution of the cacophony IVS6 region in sandflies. Insect Mol Biol. 2002;11:117–22. 10.1046/j.1365-2583.2002.00315.x 11966876

